# The Selective Advantage of the *lac* Operon for *Escherichia coli* Is Conditional on Diet and Microbiota Composition

**DOI:** 10.3389/fmicb.2021.709259

**Published:** 2021-07-21

**Authors:** Catarina Pinto, Rita Melo-Miranda, Isabel Gordo, Ana Sousa

**Affiliations:** ^1^CEDOC, Chronic Diseases Research Centre, NOVA Medical School, Faculdade de Ciências Médicas, Universidade NOVA de Lisboa, Lisbon, Portugal; ^2^Department of Medical Sciences, Institute of Biomedicine (iBiMED), University of Aveiro, Aveiro, Portugal; ^3^Instituto Gulbenkian de Ciência, Oeiras, Portugal

**Keywords:** *lac* operon, fitness effect, *Escherichia coli*, *Lactobacillus murinus*, *Bacteroides thetaiotaomicron*, lactose, gut microbiota, microbe–microbe interactions

## Abstract

The *lac* operon is one of the best known gene regulatory circuits and constitutes a landmark example of how bacteria tune their metabolism to nutritional conditions. It is nearly ubiquitous in *Escherichia coli* strains justifying the use of its phenotype, the ability to consume lactose, for species identification. Lactose is the primary sugar found in milk, which is abundant in mammals during the first weeks of life. However, lactose is virtually non-existent after the weaning period, with humans being an exception as many consume dairy products throughout their lives. The absence of lactose during adulthood in most mammals and the rarity of lactose in the environment, means that the selective pressure for maintaining the *lac* operon could be weak for long periods of time. Despite the ability to metabolize lactose being a hallmark of *E. coli*’s success when colonizing its primary habitat, the mammalian intestine, the selective value of this trait remains unknown in this ecosystem during adulthood. Here we determine the competitive advantage conferred by the *lac* operon to a commensal strain of *E. coli* when colonizing the mouse gut. We find that its benefit, which can be as high as 11%, is contingent on the presence of lactose in the diet and on the presence of other microbiota members in the gut, but the operon is never deleterious. These results help explaining the pervasiveness of the *lac* operon in *E. coli*, but also its polymorphism, as *lac*-negative *E. coli* strains albeit rare can naturally occur in the gut.

## Introduction

The *lac* operon, first described by Jacob and Monod (1961), codes for the cellular machinery to transport and metabolize lactose. It is constituted by three structural genes: *lacZ* codes for the lactose-degrading enzyme β-galactosidase, that breaks down lactose into glucose and galactose; *lacY* encodes the β-galactoside permease, that facilitates lactose transport into the cell; and *lacA* encodes galactoside transacetylase, whose function in lactose metabolism remains unknown (Müller-Hill, 1996b; Roderick, 2005). The *lac* operon is expressed under the presence of lactose and low levels of glucose (Müller-Hill, 1996a), being the textbook example of gene regulation at the transcriptional level in prokaryotes. As it is present in the vast majority of *Escherichia coli* strains (Stoebel, 2005), the phenotype of lactose consumption was used to identify *E. coli* among environmental samples (Hartl and Dykhuizen, 1984).

Lactose is the primary sugar in milk (Holsinger, 1988) and abundant in the mammalian gut during breastfeeding. However, lactose is virtually absent after that period. In fact, mammals become lactose-intolerant in adulthood, except for about a third of the human population, which exhibits lactase persistence into adulthood resulting from selection in dairy farming cultures (Storhaug et al., 2017). Thus, given the absence of lactose during adulthood in most mammals and also its scarcity in the environment, the selective pressure for preserving the *lac* operon could be weak. Yet, β-galactosidase can also degrade other compounds, including galactosylglycerols, which result from the pancreatic degradation of galactolipids (Egel, 1979, 1988). As galactolipids are main components of the chloroplast membranes, the frequent ingestion of green leaves could act as a selective pressure to maintain the *lac* operon in *E. coli* beyond the breastfeeding period. Studies on the origin of the operon were not able to trace its occurrence to a horizontal gene transfer event (Stoebel, 2005), further supporting a continuous pressure to keep the operon and the hypothesis of it being an important component of the species identity.

*E. coli*’s primary habitat is the mucus layer of the mammalian intestinal tract, where it grows in a multi-species biofilm and accounts for around 0.1% of the human gut microbiota population (Raman et al., 2005). *E. coli*, together with streptococci, is one of the first bacteria to colonize the intestinal track, being established as soon as 48 h after birth (Mackie et al., 1999). The large amounts of lactose present in the gut at this stage might confer *E. coli* an advantage for colonizing this environment (Ochman et al., 2000; Blount, 2015).

Still, the actual selective value of the *lac* operon for *E. coli* in this niche, whether it increases in the presence of lactose, and how it changes according to microbiota composition, remains undetermined. To tackle these questions, we determined the competitive advantage given by the *lac* operon to a commensal strain of *E. coli* when colonizing the mouse intestine both in the presence and absence of lactose. We found that the ability to consume lactose is mainly beneficial in the presence of lactose and that this benefit can have an individualized effect, possibly reflecting differences in microbiota composition.

To disentangle the effect of lactose from the effect of the microbiota we next performed similar competitions in animals devoid of microbiota [germ free (GF) mice]. To our surprise, the *lac* operon was neutral in this scenario, irrespectively of the presence of lactose. We hypothesized that the overabundance of nutritional sources and the lack of interspecies competition could be responsible for this result. We corroborate this hypothesis via a series of competitions varying the level of inter and intraspecies competition and show that the selective benefit of the *lac* operon depends on the presence of lactose and also gradually increases with the number of different species present in the gut.

## Materials and Methods

### Mice

All experiments were conducted using female 6–8 weeks old C57BL6/6J mice under germ free (GF) or specific pathogen-free (SPF) conditions at the Instituto Gulbenkian de Ciência (IGC) animal facilities. Mice were individually caged and had access to water and food *ad libitum*. GF mice were bred and raised at the IGC gnotobiology facility in dedicated axenic isolators (La Calhene/ORM). Young adults were transferred into sterile ISOcages (Tecniplast) before the competition experiments. This research project was approved by both the Ethics Committee and the IGC Animal Welfare Body (license reference: A009.2018), and by the Portuguese National Entity that regulates the use of laboratory animals (DGAV—Direção Geral de Alimentacão e Veterinária; license reference: 009676). All experiments were performed following the Portuguese (Decreto-Lei n° 113/2013) and European (Directive 2010/63/EU) legislations concerning animal welfare.

### Bacterial Strains

The two *E. coli* strains used in this work were derived from *E. coli* K-12 MG1655 and were resistant to streptomycin due to the point mutation *rpsL* K43T (Str^*R*^). The two strains are isogenic except for the deletion of the *lacZ* (Δ*lacZ, lac*−), differing only in the ability to metabolize lactose. *L. murinus* was isolated from feces of SPF mice and species identity was confirmed by sequencing the whole 16S rRNA gene. *Bacteroides thetaiotaomicron* strain (VPI-5482) was acquired from the German Collection of Microorganisms and Cell Cultures (DSMZ 2079).

### Mouse Gut Colonizations

To colonize the mouse intestine, we used a streptomycin treated colonization model (Conway et al., 2004) under two antibiotic regimes, i.e., treatment lasted for 1 or 7 days. GF animals were treated continuously with streptomycin. SPF animals were colonized with *E. coli*, while GF mice were either mono-colonized (*E. coli*), double-colonized (*E. coli* + *L. murinus*), or triple-colonized (*E. coli* + *L. murinus* + *B. thetaiotaomicron*). In all colonization conditions, mice were given autoclaved drinking water supplemented with streptomycin (5 g/L) for 24 h.

After 4 h of starvation for water and food, animals were gavaged with 100 μL of a suspension of 10^8^ colony forming units (CFUs) of a mixture (1:1) of *lac*− and *lac* + (SPF and mono-colonization). Double and triple colonizations started with the gavage of 100 μL of a suspension of 10^8^ CFU containing equal amounts of *E. coli* and each of the other species.

Bacteria for gavage were prepared as follows: *E. coli* was grown in LB broth overnight with aeration followed by a 1:100 dilution and grown in BHI broth until reaching an optical density of 2. *L. murinus* was inoculated in De Man, Rogosa and Sharpe (MRS) broth and incubated in anaerobiosis. *B. thetaiotaomicron* was inoculated in Chopped Meat Medium with Carbohydrates and grown in anaerobiosis. All bacteria were incubated overnight at 37°C and 1 mL of each culture was centrifuged and resuspended in PBS 1x + 0.1% cysteine.

Water with streptomycin was maintained throughout the 6 days of experiment and replaced after 3 days, except for the colonization where streptomycin was only administered for 24 h before gavage. In half of the animals, the water was also supplemented with 2% lactose. This percentage was chosen to mimic to the concentration found in mice milk (Görs et al., 2009). Fecal samples were collected and weighed daily. Samples were then suspended in PBS 1x and appropriate dilutions were then inoculated in MacConkey agar supplemented with 100 μg/mL of streptomycin (for *E. coli*) and MRS agar (for *L. murinus*) or in LB agar with X-Gal and 100 μg/mL of streptomycin. *E. coli* and *L. murinus* were grown at 37°C for 18 and 24 h, respectively in aerobiosis. When grown in MacConkey, *E. coli lac* + and *lac*− strains were discriminated by colony color (red colonies are *lac* +, white colonies are *lac*−).

### Competitive Fitness Assays *in vivo* to Measure the Selective Coefficient of the *lac* Operon

To assess if the *lac* operon confers an advantage to *E. coli* in the tested conditions, total numbers and relative frequencies of the bacteria were determined by counting the number of colonies of each strain. The selection coefficient (fitness gain) per generation of the *lac* + strain *in vivo* was calculated as the slope of the linear regression of ln (freq*_*lac*__+_*/freq*_*lac*__–_*) divided by the number of generations for *E. coli* per day. The number of days considered to calculate the selection coefficient in the colonization of SPF mice (1 day of streptomycin) was 2 (*n* = 3), 3 (*n* = 1), 4 (*n* = 3), or 5 (*n* = 1), to use a time interval with a linear tendency of selection. For the remaining colonizations, the first 3 days were considered. As for the number of generations per day, we considered this value to be 18 based on the previous estimation of 80-min generation time for *E. coli* in streptomycin-treated mice (Rang et al., 1999).

### Microbiota Analysis

To characterize the effect of different streptomycin regimes in the microbiota, we analyzed the V4 region of 16S rRNA gene sequenced from fecal samples from young animals before and after antibiotic treatment (days 7/8 of the evolution experiment). 16S sequence data from animals treated with streptomycin for only 1 day is available from Frazão et al. (2019) and 16S sequence data from animals treated with continuous streptomycin is available from Barreto et al. (2020).

Raw reads were processed using QIIME2 version 2020.8 with default parameters (Bolyen et al., 2019). Deblur was used for quality filtering and denoising (Amir et al., 2017). The generated table of ASVs was then used in the R package *phyloseq* (McMurdie and Holmes, 2013) to determine the Bray-Curtis dissimilarity index, using rarefaction based on the sample with the lowest sequencing depth. Within-group distance based on Bray-Curtis was calculated in QIIME2.

The observed ASVs were also calculated in QIIME2 with *diversity core-metrics-phylogenetic* and *diversity alpha-group-significance*, also using rarefaction based on the sample with the lowest sequencing depth.

### Statistical Analysis

All graphical representations were performed in GraphPad Prism 8.2.1 for Mac^1^ (GraphPad Software, San Diego, California, United States) and statistical analysis in Prism or in R version 3.6.3 (R Core Team, 2020). Normal data distribution was assessed using the Shapiro-Wilk test and QQ plots observation, and the homogeneity of variances using Levene’s test. Statistical significance was defined for *p* < 0.05. Significance levels were defined as: ^∗^*p* < 0.05, ^∗∗^*p* < 0.01, ^∗∗∗^*p* < 0.001, ^****^p < 0.0001. The detailed statistics and sample size for each experiment are described in the figure legends.

## Results

### The Selective Advantage of the *lac* Operon Is Stronger on the Presence of Lactose and Reflects the Amount of Competition

The selective advantage conferred by the ability to metabolize a given nutrient is expected to change with its relative abundance, its energetic potential, and the amount of competition for that nutrient.

Here we evaluated the selective advantage conferred by the *lac* operon to an *E. coli* strain (*lac* +), by allowing for its competition with a *lac*− strain in the intestine of mice after a short treatment (1 day) with streptomycin. This procedure maintains a complex microbiota while allowing for *E. coli* colonization (Frazão et al., 2019). We started by colonizing 8 animals with a mixture of 1:1 (*lac* + and *lac*−) isogenic strains, except for the deletion of the *lacZ* in the *lac-* strain, by oral gavage. We further supplemented the diet of 4 animals with 2% lactose whereas the remaining 4 animals were fed regular chow. The frequencies of *lac* +/*lac*− strains were assessed by daily collecting fecal samples and plating appropriate dilutions for 6 days.

**FIGURE 1:**
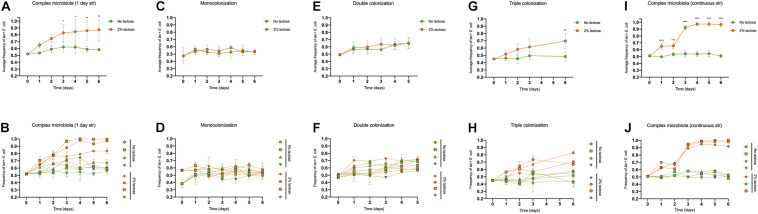
The advantage of the *lac* operon is dependent on diet and the microbiota composition. Frequency of *lac* + *E. coli* when the diet is supplemented or not with lactose, during the first 6 days of colonization of the mouse gut, with microbiotas of different complexity: **(A,B)** complex microbiota (SPF mice) after 1 day of streptomycin (*n* = 4 per group); **(C,D)** monocolonization with *E. coli* (*n* = 4 per group); **(E,F)** double colonization with *E. coli* and *L. murinus* (*n* = 4 per group); **(G,H)** triple colonization with *E. coli*, *L. murinus* and *B. thetataiomicron* (*n* = 4 per group); **(I,J)** complex microbiota (SPF mice) with continuous streptomycin treatment (*n* = 3–4 per group). In the top panels **(A,C,E,G,I)**, values are represented as mean ± SD. In the bottom panels, each animal is individually represented, and the error bars correspond to experimental replicates (*n* = 3). The effects of the microbiota composition and time on the *lac* + *E. coli* frequency were tested by a linear mixed model followed by a type III ANOVA and *post hoc* contrasts with Bonferroni’s correction.

**FIGURE 2:**
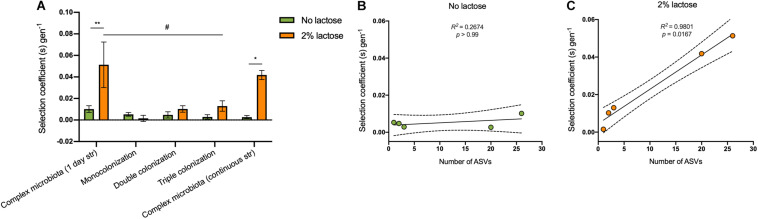
The selective advantage provided by the *lac* operon in the presence of lactose increases with microbiota complexity. **(A)** Selection coefficient per generation of the *lac* + *E. coli* strain when the diet is supplemented or not with lactose, with microbiotas of different complexity: Complex microbiota (SPF mice) after 1 day of streptomycin (*n* = 4 per group); Monocolonization with *E. coli* (*n* = 4 per group); double colonization with *E. coli* and *L. murinus* (*n* = 4 per group); triple colonization with *E. coli*, *L. murinus* and *B. thetataiomicron* (*n* = 4 per group); complex microbiota (SPF mice) with continuous streptomycin treatment (*n* = 3–4 per group). Values are represented as mean ± SEM. The effects of the microbiota composition and lactose on the selection coefficient were tested by a Two-Way ANOVA followed by *post hoc* selected contrasts with Bonferroni’s correction. **(B,C)** Linear regression of the number of ASVs vs. the selection coefficient per generation in the **(B)** absence and **(C)** presence of lactose. *P*-values refer to the significance of the Spearman’s correlation. The dashed lines represent the 95% confidence intervals. *p < 0.05, **p < 0.01 refers to comparisons between the presence or absence of lactose within each microbiota composition. ^#^*p* < 0.05 refers to comparisons between the different microbiota compositions in the presence of lactose.

During this period, in the absence of lactose, the frequency of *lac* + remained close to the initial 50% in two animals and increased slightly in the other two (Figures 1A,B), showing a mean advantage of 1 ± 0.6% (SEM) (Figure 2).

The presence of lactose had a significant effect in the *lac* + frequency over time [Figures 1A,B, *F*(6, 36) = 5.63, *p* = 0.0003] and a more variable effect was observed for the selective advantage of the *lac* + strain, ranging from 1.4 to 11%, with a mean of 5 ± 4% (SEM) (Figure 2).

Besides the presence of lactose, differences between measurements likely reflect the common variation in microbiota composition between individuals which is known to further increase with the antibiotic treatment (Leónidas Cardoso et al., 2020). The effect of the chosen antibiotic regime was assessed by estimating the Bray-Curtis beta diversity, a measure of group heterogeneity, before and after antibiotic treatment in an independent set of 5 animals. A mean increase of ∼30% in Bray-Curtis diversity was observed after antibiotic treatment (Supplementary Figures 1A,B) possibly contributing to the variation in the fitness effect of the *lac* operon in the gut of different mice.

These results led us to conclude that the *lac* operon can afford a significant advantage to an *E. coli* strain while colonizing the intestine, but its benefit is influenced by lactose and possibly microbiota composition.

To isolate the effect of lactose and test for the influence of a microbiota diversity to the selective coefficient of the *lac* operon, we engaged in a series of colonizations within a range of microbiota diversities. These comprised a mono, double and a triple colonization. A group of animals continuously treated with antibiotic (7 days) was further included, representing a complex yet simpler microbiota than the group treated only for a short period (1 day).

Although not statistically significant, after antibiotic treatment the two regimes differed in microbiota richness, with an average of 20 (continuous regime) and 26 (1 day regime) ASVs (Supplementary Figure 1C and Supplementary Table 1).

These microbiota compositions likely represent different levels of competition to *E. coli*, which could influence the abundance of lactose and other nutritional sources.

To reduce competition to a minimum we monoassociated GF animals with a mixture of 1:1 (*lac* +/*lac*−) *E. coli* and followed its frequency for a period of 6 days. As before, a group of animals received a lactose supplemented diet (*n* = 4) whereas the remaining animals were fed regular chow. Interestingly we observed that when *E. coli* is the only inhabitant of the gut, the ability to metabolize lactose, irrespectively of the presence of its cognate substrate, is a neutral trait (Figures 1C,D, 2).

Though it could be hypothesized that in this simple environment the available glucose could preclude the expression of the *lac* operon by catabolite repression this seems unlikely since glucose is less abundant in the cecum of monocolonized than in SPF mice (Barroso-Batista et al., 2020), where the *lac* operon is presumably expressed.

We proceeded by co-colonizing GF animals with *E. coli* and similar numbers of a representative species of Firmicutes, *Lactobacilus murinus*, which belongs to lactic acid bacteria, thus can compete for lactose (de Vos and Vaughan, 1994). As before, the *lac* operon conferred no particular advantage both in the presence or absence of lactose (Figures 1E,F, 2).

Therefore, to further increase the competition level, we performed a triple colonization of GF animals with equal amounts of *E. coli*, *L. murinus*, and *Bacteroides thetaiotaomicron*, which is a representative species of the phylum Bacteroidetes, usually present in large numbers in the gut microbiota and that can also compete for lactose (Chia et al., 2020).

The addition of a third species was enough to restore the advantage of the *lac* operon in the presence of lactose [Figures 1G,H, *F*(4, 24) = 4.41, *p* = 0.0082], providing a selective advantage around 1.3 ± 1% (SEM) per generation (Figure 2). Nevertheless, we cannot rule out the possibility that *B. thetaiotaomicron per se*, and not just the number of species, was responsible for the increase in the effect of the *lac* operon since we did not perform a colonization including only *E. coli* and *B. thetaiotaomicron*.

Coherently, in the context of continuously antibiotic treated mice and in the presence of lactose, the effect of the *lac* operon was further increased showing a mean advantage of 4.2 ± 1% per generation (Figure 2) which allowed it to reach a frequency close to fixation by day 4 (Figures 1I,J). Interestingly, the variation in *lac* + selective coefficient between animals was also reduced in comparison to the 1 day antibiotic regime (Figure 2), in agreement with the smaller effect of the antibiotic in group heterogeneity in the continuous regime [Supplementary Figures 1A,B, *p* = 0.0007 (Kruskal-Wallis test, *post hoc* comparisons with Dunn’s correction)].

Globally, these experiments demonstrate that the benefit of the *lac* operon is potentiated by the presence of lactose and increases with the number of co-colonizers (Figures 2B,C).

### The Absolute Success of *E. coli* in the Gut Depends on the Net Result Between Synergism and Competition

Increasing microbiota diversity likely promotes competition but it also raises the probability of synergism, e.g., via cross-feeding.

**FIGURE 3:**
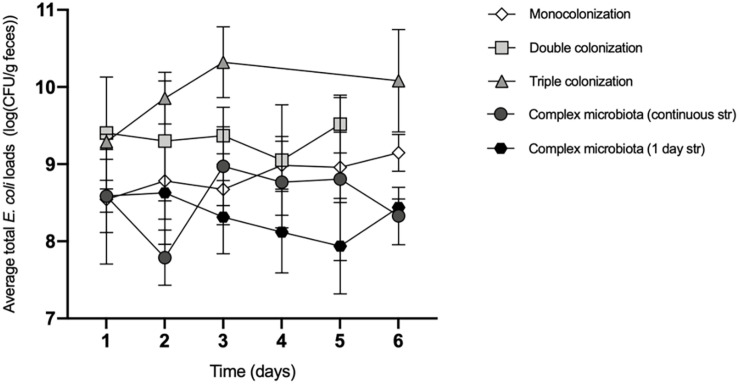
The total *E. coli* loads vary with the composition of the microbiota. Average *E. coli* total loads (log_10_ transformed) during the first 6 days of colonization of the mouse gut, with increasing microbiota complexity: Monocolonization with *E. coli*, *n* = 8; Double colonization with *E. coli* and *L. murinus*, *n* = 6; Triple colonization with *E. coli*, *L. murinus* and *B. thetataiomicron*, *n* = 8; Complex microbiota with continuous streptomycin treatment, *n* = 7; Complex microbiota after 1 day of streptomycin, *n* = 8. Values are represented as mean ± SD. The effects of the microbiota composition and time on the total *E. coli* loads were tested by a linear mixed model followed by type III ANOVA. *Post hoc* comparisons were performed between all the levels of microbiota complexity for day 5 and 6 (Bonferroni’s correction). In day 5, *E. coli* loads in the complex microbiota (continuous streptomycin) are significantly lower than: the monocolonization (*p* = 0.0036); double colonization (*p* < 0.0001); and the complex microbiota (continuous str) (*p* = 0.038). In day 6, *E. coli* loads in the triple colonization are higher than the monocolonization (*p* = 0.0117) and the complex microbiota (both 1 day and continuous str) (*p* < 0.0001).

Here we observe that *E. coli*’s abundance (measured by its loads—CFU/g of feces) increases with the number of co-colonizers in ex-GF mice, but then it drops when the gut environment reaches the complexity of a regular microbiota (Figure 3). Specifically, when comparing the double with the mono colonization we observed a non-significant but sustained increase in the average loads of *E. coli* in the double colonization in 4 out of the 5 days of the experiment (Figure 3). Upon 5 days of colonization *E. coli* was on average 3 times more abundant in the double (∼4.4 × 10^9^ CFU/g) than in the mono colonization (∼1.3 × 10^9^ CFU/g).

The sharpest increase was observed when comparing the mono with the triple colonization where a significant difference (∼10 times) in the average loads of *E. coli* was measured (2 × 10^10^ and 1.6 × 10^9^ CFU/g at the 6th day of the experiment, in the triple and mono colonization, respectively (*p* = 0.012, *post hoc* comparison with Bonferroni’s correction).

These data suggest that *E. coli*’s niche is expanded by the presence of *L. murinus* and *B. thetaiotaomicron*, hinting at a synergistic relationship between these bacteria and *E. coli*.

In contrast, the expansion of *E. coli* in an environment with multiple species of bacteria, such as the microbiota of streptomycin-treated mice, regardless of the antibiotic regime, was limited to ∼10^8^ CFU/g upon 6 days of colonization, likely due to the predominance of interspecies competition.

These differences are similar for the *lac* + and *lac*− strains separately (Supplementary Figure 2) and are independent of the presence of lactose in the mouse diet (Supplementary Figure 3).

## Discussion

The nearly ubiquitous presence of the *lac* operon in *E. coli* and the restricted timeframe of lactose availability in its natural environment, the mammalian gut, has for long raised questions about the adaptive value of the *lac* operon in *E. coli.* Breastfeeding represents only a brief period in mammalians’ life, but it is particularly influential in determining the first stages of microbiota establishment (Milani et al., 2017), possibly with long-term effects. *E. coli* represents a small fraction of the adult microbiota (Raman et al., 2005), but is among the first colonizers of the gut (Milani et al., 2017) and therefore, consuming lactose, could in principle, contributes to its success. In contrast, it has it has been shown that lactose consumption is not essential for *E. coli* to colonize the intestine of streptomycin-treated SPF CD-1 mice, while essential sugars for either the initiation or maintenance of gut colonization are arabinose, fucose, gluconate, N-acetyl-glucosamine, and N-acetyl-neuraminic acid (Fabich et al., 2008).

In our work, by allowing for direct competition between *lac* + and *lac− E. coli* strains in the adult mammalian gut we found that, in the presence of lactose, the *lac* operon confers a selective advantage that can be as high as 11%, while being close to neutral in its absence. Therefore, our results suggest that the strong interspecies competition for nutritional resources that occurs in these settings (Coyte et al., 2015) considerably increases the advantage for lactose consumers, particularly in the presence of lactose.

The emergence of constitutive mutants for lactose consumption during the first days of life was observed in a controlled experiment (Ghalayini et al., 2019), further supporting the hypothesis that the *lac* operon is under strong selection during this period, which in the long term could help *E. coli* to secure a place in the complex adult microbiota.

Besides the presence of lactose, the fitness advantage of the *lac* operon for *E. coli* was also shown to depend on microbiota composition, particularly on the number of species present. One way to interpret this result is to consider that an increasing number of species increases the opportunity for more competitive interactions. Thus, the ability to consume lactose would become a bigger advantage as other nutritional sources get depleted by the growing number of competitors. On the other hand, as the number of species increases, so does the opportunity for synergism, with the absolute success of *E. coli* reflecting the net result between these two forms of ecological interactions.

The observation that the population size of *E. coli* substantially increased in the triple-colonization, in comparison to the situation where it is alone (mono-colonization), showed us that, from the perspective of *E. coli*, synergism dominated in this scenario. This is consistent with the notion that *E. coli* consumes the byproducts of complex nutrients’ degraded by *B. thetaiotaomicron*, such as maltose and fucose which result from the breakdown of dietary glycans (Chang et al., 2004; Li et al., 2015; Baümler and Sperandio, 2016). This seems to occur regardless of the ability to metabolize lactose, and it is independent of the availability of that additional carbohydrate.

In contrast to our findings, in a scenario of DSS-induced colitis *E. coli* was antagonized by *B. thetaiotaomicron* and another species of *Lactobacillus* (*L. johnsonii*). Here the oral administration of these species was enough to reduce the overgrowth of *E. coli* (Charlet et al., 2020). The higher microbiota complexity of the animals in the referred study when compared to ours, as well as potentially different strains, may account for this difference.

Conversely, in the presence of a complex microbiota (several tens of species), its absolute success decreased and the most likely scenario was one dominated by competition (Coyte et al., 2015). A scenario where the population size of *E. coli* in the gut is inversely correlated with microbiota diversity has been previously observed in more than one occasion (Sousa et al., 2017).

Surprisingly, when comparing the mono with the triple colonization, lactose showed to be more advantageous in the situation where *E. coli*’s absolute success was higher, suggesting that the fitness effect of the *lac* operon may be density dependent. Future work should address this issue, possibly through investigating the geographic distribution of bacteria abundances and possible differences in the *lac* operon selective advantage along the intestinal tract. Since lactose is mainly absorbed in the small intestine, this could be the place where *lac* + is positively selected with the cecum being the intestinal compartment where *E. coli* expands irrespectively of its ability to consume lactose. Such a hypothetical situation would simultaneously explain the larger advantage of a *lac* + strain and the higher absolute success of the species.

Another interesting observation was the correlation between the variance of the *lac* operon selective effect and microbiota heterogeneity. This was unraveled by the two antibiotic regimes but probably best resemble the natural diversity of the microbiota of animals which are not genetically homogenous or eat the same controlled diet as in our experimental setup.

Overall, the results indicate that the specificity of interactions established by *E. coli*, and, therefore, the balance between beneficial and antagonistic interactions, determines its niche size and location. The adaptive value of the *lac* operon for *E. coli* is dependent on the competitors and the availability of nutritional resources and might be especially useful in highly competitive environments and for exploring new niches.

## Data Availability Statement

The original contributions presented in the study are included in the article/Supplementary Material, further inquiries can be directed to the corresponding author/s.

## Ethics Statement

The animal study was reviewed and approved by Direção Geral de Alimentacão e Veterinária (license reference: 009676) and Instituto Gulbenkian de Ciência Animal Welfare Body (license reference: A009.2018).

## Author Contributions

CP, IG, and AS conceived and designed the experiments. CP performed the experiments. CP, RM-M, and AS analyzed the data. IG and AS contributed reagents, materials, and analysis tools. RM-M and AS wrote the manuscript. CP and IG contributed to the writing of the manuscript. All authors contributed to the article and approved the submitted version.

## Conflict of Interest

The authors declare that the research was conducted in the absence of any commercial or financial relationships that could be construed as a potential conflict of interest.

## Footnotes

1 www.graphpad.com
