# Corrigendum: The selective advantage of the *lac* operon for *Escherichia coli* is conditional on diet and microbiota composition

**DOI:** 10.3389/fmicb.2023.1332365

**Published:** 2023-11-27

**Authors:** Catarina Pinto, Rita Melo-Miranda, Isabel Gordo, Ana Sousa

**Affiliations:** ^1^CEDOC, Chronic Diseases Research Centre, NOVA Medical School, Faculdade de Ciências Médicas, Universidade NOVA de Lisboa, Lisbon, Portugal; ^2^Department of Medical Sciences, Institute of Biomedicine (iBiMED), University of Aveiro, Aveiro, Portugal; ^3^Instituto Gulbenkian de Ciência, Oeiras, Portugal

**Keywords:** *lac* operon, fitness effect, *Escherichia coli*, *Lactobacillus murinus*, *Bacteroides thetaiotaomicron*, lactose, gut microbiota, microbe–microbe interactions

In the published article, there was an error in the Funding statement. The funding statement was displayed as “This work was funded by the Fundação para a Ciência e Tecnologia (PTDC/BIA-EVF/3247/2012 and PTDC/BIA-EVL/30212/2017) and iBiMED (UID/BIM/04501/2019, project POCI-01-0145-FEDER-007628).”. The correct Funding statement appears below.

