# Supplementary material for: The Selective Advantage of the lac Operon for Escherichia coli Is Conditional on Diet and Microbiota Composition
Source: Front Microbiol. 2021 Jul 21;12:709259. doi: 10.3389/fmicb.2021.709259 (PMC8333865; doi:10.3389/fmicb.2021.709259)
Supplement: Supplementary file 1 [file Image_1.pdf]

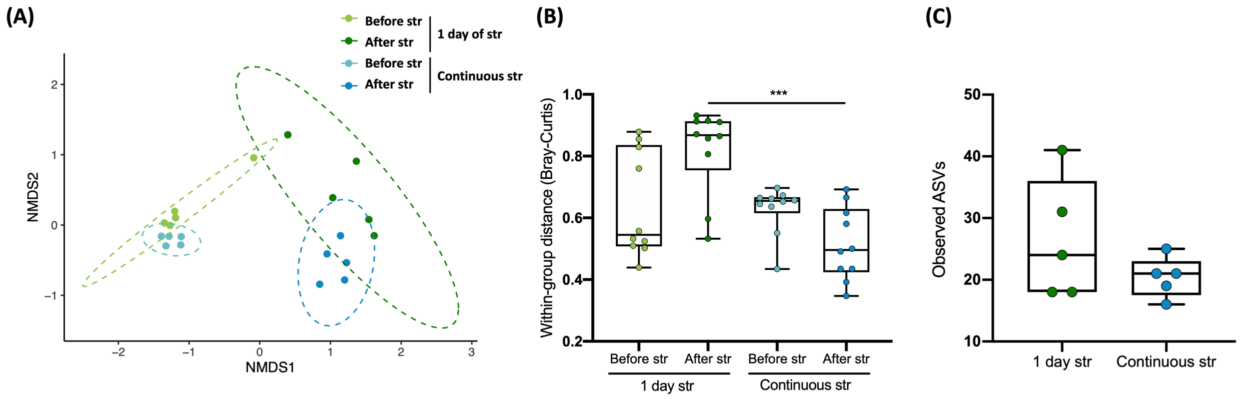

Supplementary Figure 1. The increase in microbiota heterogeneity after streptomycin administration depends on antibiotic regime. (A) Non-metric multi-dimensional scaling **(**NMDS) and (B) within-group distance based on Bray-Curtis dissimilarity index, comparing the effect of streptomycin in the two antibiotic regimens (1 day *vs.* continuous administration). Significant differences were tested with the Kruskal-Wallis test followed by post-hoc comparisons with Dunn's correction. (C) Richness (observed ASVs) after the two antibiotic treatments. Significant differences were tested with one-tailed Mann-Whitney test.
