# Supplementary material for: The Selective Advantage of the lac Operon for Escherichia coli Is Conditional on Diet and Microbiota Composition
Source: Front Microbiol. 2021 Jul 21;12:709259. doi: 10.3389/fmicb.2021.709259 (PMC8333865; doi:10.3389/fmicb.2021.709259)
Supplement: Supplementary file 2 [file Image_2.PDF]

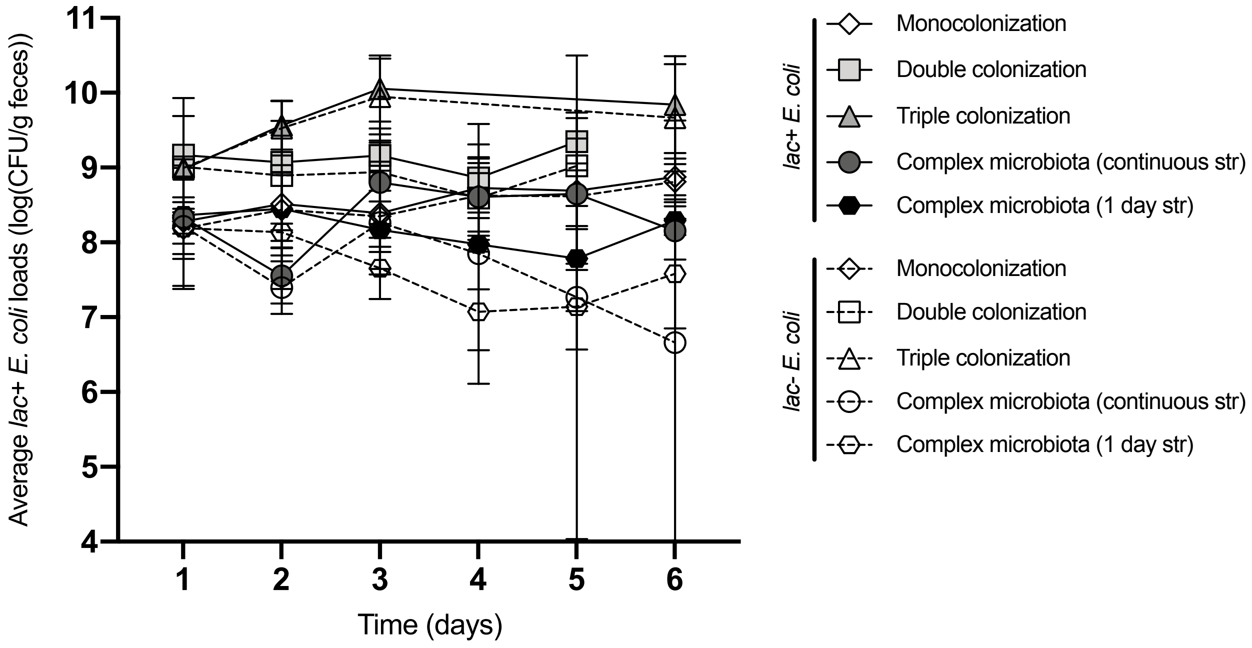

Supplementary Figure 2. The *E. coli* loads are independent of the ability to metabolize lactose. *Lac*+ and *lac*- *E. coli* loads (log_{10} transformed) during the first 6 days of colonization of the mouse gut, with increasing microbiota complexity: Monocolonization with *E. coli*, *n*=8; Double colonization with *E. coli* and *L. murinus*, *n*=6; Triple colonization with *E. coli*, *L. murinus* and *B. thetataiomicron*, *n*=8; Complex microbiota with continuous streptomycin treatment, *n*=7; Complex microbiota after 1 day of streptomycin, *n*=8. Values are represented as mean ± SD.
