# Supplementary material for: The Selective Advantage of the lac Operon for Escherichia coli Is Conditional on Diet and Microbiota Composition
Source: Front Microbiol. 2021 Jul 21;12:709259. doi: 10.3389/fmicb.2021.709259 (PMC8333865; doi:10.3389/fmicb.2021.709259)
Supplement: Supplementary file 3 [file Image_3.pdf]

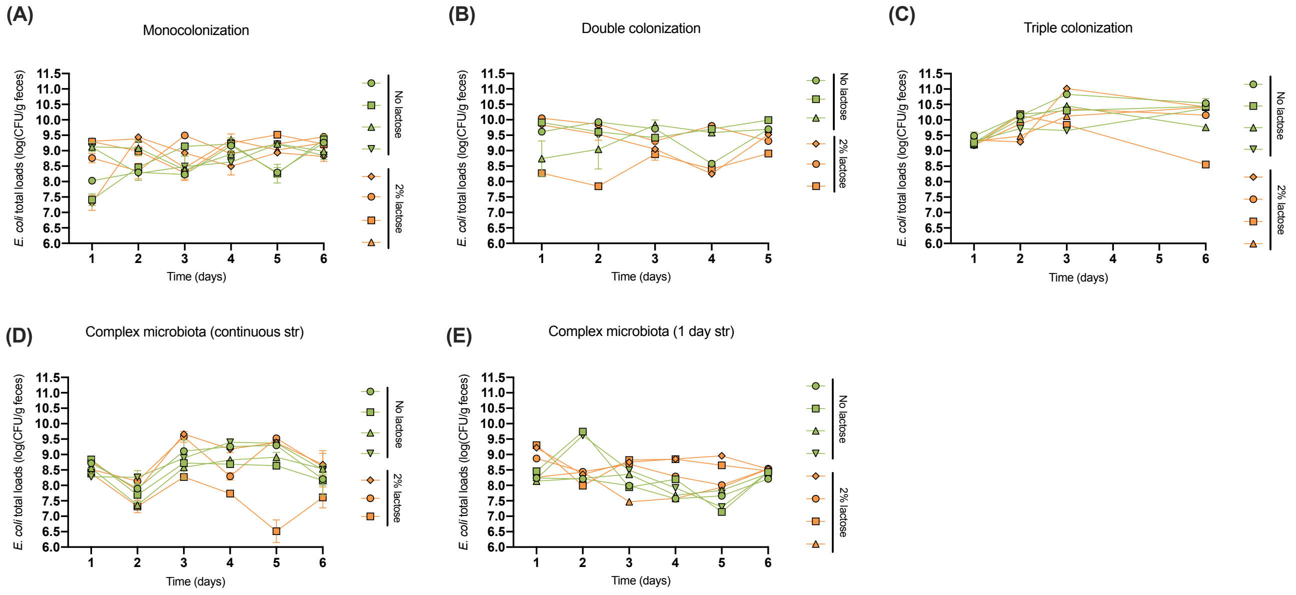

Supplementary Figure 3. The *E. coli* total loads are not dependent on the presence of lactose. *E. coli* total loads (log_{10} transformed) per animal during 5 or 6 days of colonization, in the absence or presence of lactose in (A) Monocolonization with *E. coli*; (B) Double colonization with *E. coli* and *L. murinus*; (C) Triple colonization with *E. coli*, *L. murinus* and *B. thetataiomicron*; (D) Complex microbiota with continuous streptomycin treatment; (E) Complex microbiota after 1 day of streptomycin, *n*=8. Green lines correspond to no lactose supplementation and orange lines to 2% lactose supplementation. Each point corresponds to the average of 3 experimental replicates ± SD.
